# Genome and characteristics of *Arthrobacter globiformis* AZ cluster phage London

**DOI:** 10.1128/MRA.00819-23

**Published:** 2023-10-31

**Authors:** Andrea R. Beyer, Hannah L. Hatke Hughes, Jasmae S. Flowers, Jacquelin Bullock, Demesha T. Daniels, Makayla Drew, Queen Lee-Mayes, Brianna M. Marshall, Vivica Remson, Micaelah Thomas

**Affiliations:** 1 Department of Biology, Virginia State University, Petersburg, Virginia, USA; DOE Joint Genome Institute, Berkeley, California, USA

**Keywords:** bacteriophages, arthrobacter, genomes

## Abstract

London is a predicted temperate bacteriophage with siphovirus morphology infecting *Arthrobacter globiformis* NRRL strain B-2880. Sequencing of the genome revealed a length of 43,599 bp comprising 69 predicted open-reading frames and no tRNA genes. It is categorized as a cluster AZ1 phage along with closely related actinobacteriophages Elezi, Eraser, and Niobe.

## ANNOUNCEMENT

**Fig 1:**
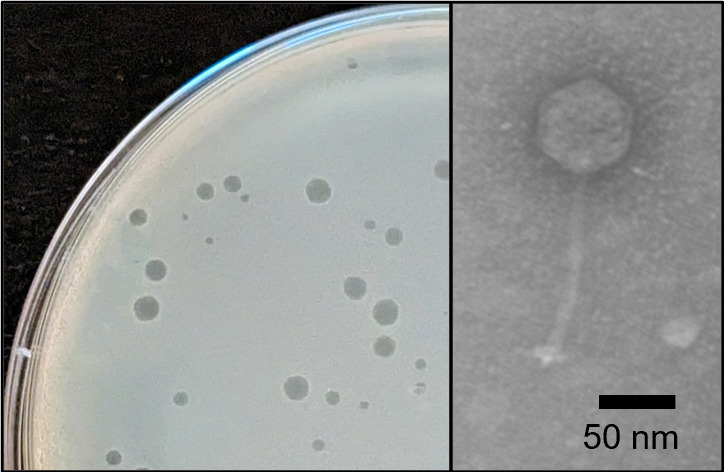
*Arthrobacter* phage London. (Left) Plaques with turbid edges and clear centers (1–3 mm in diamter) formed after 24 h of incubation at 30°C on peptone-yeast extract calcium medium with host *Arthrobacter globiformis* NRRL B-2880. Plaques became more turbid after 48 h. (Right) Negative-staining transmission electron micrograph of London, a siphovirus with a tail length of 114–121 nm (*n* = 17) and an isometric capsid of 55–62 nm in diameter (*n* = 20). Scale bar is 50 nm. Imaged on a Philips CM-10 TEM at 80 kV.

In an age where antibiotic resistance is becoming a significant healthcare threat, bacteriophages offer an alternative treatment for managing bacterial infections. Additionally, novel infections are emerging with ubiquitous and previously “benign” bacteria such as soil-borne *Arthrobacter* species (1). Here, we report on the discovery of phage London, isolated from soil collected near Chesapeake, Virginia, USA (36.780000N, 76.275000W) in September 2019 using standard methods (2). Briefly, collected soil was shaken in peptone-yeast extract calcium medium for 1 h, and the supernatant was passed through a 0.2-µm filter. Filtrate was inoculated with *Arthrobacter globiformis* NRRL B-2880 and incubated with shaking for 48 h at 30°C prior to further filtration and plating using a 0.4% top agar overlay with *A. globiformis*. Plates were incubated at 30°C, and resulting plaques (Fig. 1, left) were purified through three rounds of plating. Negative staining with 1% uranyl acetate and transmission electron microscopy revealed London as having siphovirus morphology (Fig. 1, right). Genomic DNA was isolated from London lysates using a Promega Wizard DNA cleanup kit, and sequencing libraries were created with NEB Ultra II Library Kit (v.3) reagents. DNA sequencing was completed on an Illumina MiSeq platform, with 829,566 single-end 150-bp reads and approximately 2,712-fold coverage. Sequences were assembled and finished as previously described (3) using Newbler (v.2.9) with default parameters and Consed (v.29).

London’s genome is 43,599 bp long with 3′ 11-bp single-stranded ends. It has 66.6% G-C content, like that of the host used, *Arthrobacter globiformis* [66% G-C (4)]. Based on >50% nucleotide similarity with previously sequenced phages (5), London was classified as a cluster AZ1 phage in the PhagesDB Actinobacteriophage Database (6). Putative genes were identified using GLIMMER (v.3.02) (7), GeneMarkS (v.2.5) (8), Phamerator (9), and Starterator (v.1.0.1) (https://github.com/SEA-PHAGES/starterator). National Center for Biotechnology Information BLASTP [nonredundant protein database (10)], HHpred [default databases (11)], and the Conserved Domain Database (12) were used to predict functions. Transmembrane proteins were evaluated with TMHMM (v.2.0; now DeepTMHMM, https://services.healthtech.dtu.dk/service.php?DeepTMHMM), and SOSUI (13). ARAGORN (v.1.2.38) (14) and tRNAscan-SE (v.2.0) (15) did not reveal any putative tRNA or transfer messenger RNAs.

Annotation data were compiled using PECAAN (https://discover.kbrinsgd.org) and DNA Master (v.5.23.3) [cobamide2.bio.pitt.edu (16)]. Default settings were used for all software. A total of 69 genes were identified, 35 of which cannot be assigned a predicted function. All but two of London’s genes are transcribed in the forward direction. The left half of the genome contains conserved structural and assembly genes, while the remainder of the genome contains genes with various hypothesized enzymatic functions supporting DNA replication and a predicted temperate lifestyle, including a serine integrase. The overall genome structure of London is consistent with those of other AZ1 phage, including the right half of the genome consisting of multiple small genes of no known function.

## Data Availability

London is available at GenBank with accession number MT889366 and Sequence Read Archive number SRX20916067.
